# 'You give us rangoli, we give you talk': using an art-based activity to elicit data from a seldom heard group

**DOI:** 10.1186/1471-2288-12-7

**Published:** 2012-01-30

**Authors:** Sabi Redwood, Nicola K Gale, Sheila Greenfield

**Affiliations:** 1 University of Birmingham, Edgbaston Campus, Birmingham B15 2TT, UK

## Abstract

**Background:**

The exclusion from health research of groups most affected by poor health is an issue not only of poor science, but also of ethics and social justice. Even if exclusion is inadvertent and unplanned, policy makers will be uninformed by the data and experiences of these groups. The effect on the allocation of resources is likely to be an exacerbation of health inequalities.

**Discussion:**

We subject to critical analysis the notion that certain groups, by virtue of sharing a particular identity, are inaccessible to researchers - a phenomenon often problematically referred to as 'hard to reach'. We use the term 'seldom heard' to move the emphasis from a perceived innate characteristic of these groups to a consideration of the methods we choose as researchers. Drawing on a study exploring the intersections of faith, culture, health and food, we describe a process of recruitment, data collection and analysis in which we sought to overcome barriers to participation. As we were interested in the voices of South Asian women, many of whom are largely invisible in public life, we adopted an approach to data collection which was culturally in tune with the women's lives and values. A collaborative activity mirroring food preparation provided a focus for talk and created an environment conducive to data collection. We discuss the importance of what we term 'shoe leather research' which involves visiting the local area, meeting potential gatekeepers, and attending public events in order to develop our profile as researchers in the community. We examine issues of ethics, data quality, management and analysis which were raised by our choice of method.

**Summary:**

In order to work towards a more theoretical understanding of how material, social and cultural factors are connected and influence each other in ways that have effects on health, researchers must attend to the quality of the data they collect to generate finely grained and contextually relevant findings. This in turn will inform the design of culturally sensitive health care services. To achieve this, researchers need to consider methods of recruitment; the makeup of the research team; issues of gender, faith and culture; and data quality, management and analysis.

## Background

Despite their greater burden of disease, people from a South Asian background and other minority ethnic communities are underrepresented in clinical and applied health research [1-3] and their recruitment to and successful participation in studies remain problematic [4]. The issues of recruitment and participation of minority ethnic groups pose challenges not only for researchers conducting randomised control trials and cohort studies, but also for qualitative researchers in applied health research [5-7].

In this article, we set out some of the underlying reasons for the underrepresentation of minority ethnic groups in clinical and health research, and discuss how methods of recruitment and data collection can inadvertently serve to exclude them, rendering them what has been termed 'hard to reach'. We draw on a study based in Birmingham in the United Kingdom in which we explored South Asian women's experience of food preparation, and living with and cooking for family members with type 2 diabetes. We were particularly concerned with what Crotty [8] has termed the pre-swallowing domain of behaviour, culture, society and experience which she contrasts with the post-swallowing world of biology, physiology, biochemistry and pathology. Our aim was to find out how eating practices are embedded in social and cultural contexts and in the flow of daily life [9]. In South Asian communities it is women who bear the responsibility for cooking and food preparation. Yet their voices have been largely missing from the health research literature, despite their potentially valuable contribution to knowledge about cooking practices, and the intersection of faith, culture, health and food. We describe an approach of doing research with this particular group which we developed in collaboration with a local artist, in order to create an appropriate environment for the research participants and to provide stimuli for discussion in an effort to gain accounts of experience, views and perspectives, memories and meanings. Although this approach was particularly suitable for this group, we are not advocating the use of Rangoli as a technique to gather data from all those who are underrepresented in research. Rather, we set out the need to adopt creative alternatives to eliciting data which are meaningful to a particular group. This may involve activities and settings not usually associated with research in order to generate evidence for addressing cultural difference in the design and provision of services. Much like a 'bricoleur, a term Lévi-Strauss [10] coined to describe how the use of an object, or in this case an activity, is reconceived to explore their possibilities and re-direct their purpose so that they may serve in a new setting, we used an activity known to research participants in a novel way that facilitated the collection of data. Such a novel approach is likely to involve different techniques for different groups. Finally, we discuss the implications of adopting such an approach in relation to participant recruitment, ethics, data collection, analysis, and quality, drawing out both strengths and limitations.

### The making of the 'hard-to-reach'

The exclusion of certain groups from clinical research constitutes poor science [1] in terms of the implications for the validity and generalisability of research findings [11]. However, it also contributes to health inequalities insofar as research affects the allocation of, and access to, power and resources [12]. At a time when the unequal distribution of the burden of ill-health continues to be a growing problem in the industrialised world [13-16], the exclusion of groups most affected by poor health becomes an issue not only of science, but also of ethics and social justice. Everyone has a right to participate in research, and researchers have an obligation to treat potential participants fairly and develop inclusive recruitment practices [17,18]. The participation in health research by all members of society is necessary to generate inclusive and culturally sensitive research evidence to inform the design of effective, suitable and equitable health interventions and services.

Reasons for the underrepresentation of minority groups in UK and US health research have recently been summarised Rugkåsa and Canvin [5]. They describe a range of issues which we broadly divide into three categories: deliberate, unintended, and conceptual exclusion. Failure to include people in health research due to perceived cultural or communication barriers can be described as deliberate exclusion. Such a decision may be motivated by the composition of the research team where there is an absence of members of minority ethnic groups; a lack of language, interpretation and language skills by the research team; insufficient resources in terms of time and money to facilitate and support the recruitment and participation of minority groups; and a lack of understanding of the importance of including minority groups in research studies for both scientific and ethical reasons. Lo and Garan [19] also propose that negative attitudes of researchers could influence their decision to recruit members of minority groups in their studies because they believe that those who do not speak English and might also lack, for instance, housing or transport, are likely to have difficulty in keeping appointments or complying with the study protocol. Such attitudes will clearly limit minority ethnic representation in research.

Unintended exclusion occurs when, even though minority ethnic groups were included in the design of the study, no or few people from these groups actually take part; or when despite efforts made to recruit and support their participation recruitment is low. The reasons for this are manifold and range from insufficient numbers of people from minority ethnic groups being available for recruitment to ineffective recruitment strategies and inadequate resources. Pinto et al [20] emphasise the importance of researchers to actively enhance and support the recruitment and retention of those who are underrepresented in research, such as women from minority ethnic communities who have experienced trauma and substances abuse, in order to produce findings with the potential to address health inequalities. Unintended exclusion also occurs as a result of a lack of willingness by targeted groups to take part in research. The reason is likely to lie in scepticism and distrust by minority ethnic groups of research in general, and research led by members of the majority ethnic group in particular. As Lo and Garan [19] suggest in relation to the USA, a powerful deterrent effect on research participation by black and minority ethnic groups has been the knowledge of previous research scandals, such as the Tuskegee Syphilis study which lasted over 30 years, and it was revealed in 1972 that treatment was withheld from black African American men long after it was known that syphilis could be treated with penicillin [21]. However, there have also been recent allegations of research misconduct in studies in which research subjects were largely members of minority groups, further exacerbating mistrust and suspicion of clinical research [22]. Unfavourable previous experience of involvement in research or concern about whether findings will have unwelcome repercussions for participants or their community will also lead to a lack of willingness to engage with research [23].

Conceptual exclusion is associated with unintended exclusion and refers to cultural differences in the understanding of research as well as differences in priorities and values [1,24]. We use the term conceptual exclusion more specifically to denote the situation where there is a fundamental mismatch between researchers' and potential participants' understanding of the nature of knowledge production. Researchers' underlying assumptions about what constitutes valuable knowledge may not be congruent with potential participants' views on what constitutes a question worthy of investigation and how to answer it. This is often described as a knowledge deficit, rather than as a difference in conceptualisations of knowledge and relevance. Potential participants may also feel marginalised by the very agencies requesting their participation in research and they may not see their concerns reflected in the scope of the research study. Consequently, different conceptualisations of the purpose and nature of research, different social and cultural norms, and a lack of common goals are likely to lead to non-participation by minority and already marginalised groups.

In relation to these types of exclusion, the term 'hard to reach' has become shorthand for describing people for whom the usual strategies of contact and engagement do not work. It is increasingly being used in the research literature to construct the problem of non-participation in research by certain groups in society. It has originally been linked to problems of access to services in health and social care as well as local government, and is frequently used in discourses about health inequalities [25,26]. With regard to the research literature, the problem of non-participation and absence from research has been largely presented as a social fact [27,28] and has not been subject to critical analysis. Reasons for being described as 'hard to reach' include having compelling motivations for remaining 'hidden' such as parents using illicit drugs [28,29], as well as belonging to groups such as sex workers [30], injecting drug users and men who have sex with men [31]. Groups with whom traditional, discursive interview based research is less appropriate have also been described as 'hard to reach', for example young men from travelling communities, or some people with cognitive or learning disabilities [32]. Minority ethnic groups have attracted the label too [33]. This notion that certain people, by virtue of sharing a certain identity are invisible and inaccessible by ordinary means, has become powerful and ubiquitous [34] and 'reaching' them has become somewhat of a holy grail. However, there are dangers associated with the use of such a term because it suggests homogeneity within and across groups that are described this way [26]. Multiple and various identities of different marginalised groups are thus conflated and essentialised. The term is also potentially misleading and stigmatising, and it tends to imply that the blame for being 'hard to reach' belongs with the group itself [25].

The group with whom we have been working are frequently described as 'hard to reach' because they tend not to be visible in public life. They are South Asian women, many of whom are first generation immigrants. For them the goals and methods of science are not congruent with their priorities and realities. They may be 'hard to reach' primarily in terms of research participation because of the very methods being used to recruit and to collect data from them. For example, letters of invitation addressed to a woman may be opened by the male head of household who decides whether or not it is appropriate for her to take part in research. Participation may be problematic for families adhering to strict traditional social norms where research is situated in the public realm and thus unsuitable for women to enter. Requirements for signed consent pose similar hurdles.

### South Asian women and qualitative interviews

Qualitative researchers have addressed and tackled the challenges of research recruitment and participation of those whose voices are similarly seldom heard [5,6,39,35-37]. Some have highlighted the importance of carrying out detailed formative research in a particular community before starting recruitment and employing a number of different recruitment methods depending on the communities' local ethnic identities and social networks [12,38]. Others have discussed particular methods to increase participation: for example, the use of data collection techniques based on traditional community social processes [39], the forging of close working relationships with local staff [40], interviews being carried out by 'cultural insiders' [41] the development of transcultural awareness and sensitivity [23] and accessing gatekeepers, advertising, snowball sampling and building reciprocal relationships [42].

However, we suggest that it is not only the methods of recruitment which have the potential to exclude certain groups and communities, but also the methods of qualitative research themselves. Research methods are not culturally neutral even though their assumptions are rarely discussed. One particular method continues to be the mainstay of qualitative health research: the research interview. Lawton [43] points out that interviews invite a certain kind of communication in which interviewees may be more concerned about the way they present themselves in relation to what they believe the interviewer's views are than conveying their beliefs and practices in more direct ways. Furthermore, the question-answer style of semi-structured interviews, or indeed the open-endedness of unstructured interviews, tends to be culturally alien to many first generation South Asian migrants. It may work to exclude South Asian women because the interviewee is unlikely to share the assumptions and expectations of the researcher/interviewer, even if s/he has a similar background. This is because research and research training in the biomedical and social sciences tend to have a particular model of the autonomous, rational, individual self, in control of their own lives and able to make decisions that are in their own best interests. This leads to an expectation that interviewees perform in particular ways and present accounts of themselves in particular ways [44]. Even if recruitment is successful, this lack of congruence is likely to lead to the danger of flat and one-dimensional data, i.e. data that fail to make visible the assumptions and explanations behind attitudes and experiences. Although the challenges of cross-cultural interviewing are raised [45], the effects on and implications for the quality of data are rarely addressed. We do not claim to be able to solve this problem. However, careful consideration and attention to culturally sensitive ways of facilitating expression and communication between the participant and researcher can lead to creative ways of generating useful data.

The limitations of the research interview in qualitative health research in general, not just with regard to groups and communities which have been labelled 'hard to reach', have also been acknowledged [46]. One-off interviews can be collected with relative ease by relatively junior research staff and therefore are often favoured in research grant applications. However, the lack of skills on the part of the interviewers to move beyond responses that would be perceived as socially acceptable to the researcher by the interviewee, taking interview data at face value and analysing it using tight pre-established frameworks often results in a lack of explanatory power of findings [47]. As a consequence, one-off interview based studies rarely move beyond the first, although undoubtedly crucial, step of developing descriptive categories. They are also at risk of generating findings and recommendations which tend to reflect common sense and single perspective views, rather than finely grained insights and explanations necessary to understand peoples' wider social and cultural contexts which shape the way people think about, for example, health and illness, suffering, self-care, gendered social roles and responsibilities, childrearing, and work.

New or additional insights may be gained by the use of methods which are relatively new to health research and which look to the arts, humanities and social sciences for tools which are transferred into the health care context. They can provide insights into the experience of health and illness, lay and patients' knowledge and perceptions, and views on services and interventions. They include, inter alia, 'lifegrids' to explore the course of illness experiences [48], 'walk-arounds' or 'participatory maps' to gain an in-depth understanding of context, decision making and individual experience [49], photo elicitation to investigate peoples' perceptions of 'being at risk' from developing an illness or doing physical activity, and what recovery from illness means to patients [50,51]. Although visual and arts-based approaches have been used with children and in therapeutic contexts, the usefulness of their application in health care research has not yet been established through rigorous analysis and evaluation. There is a case to be made to push qualitative approaches in health research further, in a way that is less descriptive and has more explanatory power [47]. This is important at a time when public health policy makers recognise that a focus on personal choice and responsibility in health related behaviour is insufficient to address the wider structural and material conditions in which individual risk of chronic illness is embedded. Indeed, lifestyle oriented interventions aimed at individual behaviour change which do not take account of the circumstances in which people live are unlikely to have a significant impact upon people's ability to manage chronic illness such as type 2 diabetes and are being critiqued for neglecting the wider social, cultural and economic context driving these behaviours [14]. We have been developing a pragmatic approach to methods for eliciting data that has greater potential to bring to the surface contextual, social and cultural factors in order to contribute to a more holistic view of the design of services, interventions and programmes to support people in leading healthy lives or managing chronic illness.

### Background to the study

In the UK, people of Indian, Pakistani, Bangladeshi and Sri Lankan descent are referred to as South Asian. They are the largest minority ethnic group and comprise the majority ethnic group in several urban locations [2]. South Asian communities living in the UK are diverse in terms of nationality, religious belief and cultural and social practices. The term South Asian is a blanket term which reduces to it different parameters such as national boundaries, language and religion. In doing so, it gives the illusion of homogeneity and fails to take account of the different features of identity. Within these groups there are no traditional eating practices or uniform perspectives on, for example, lifestyle, adherence to medical advice, and education. However, there is a tendency for South Asian culture to be portrayed as uniform and rigid, and as a negative influence on the self-management of type 2 diabetes and the prevention of complications [52]. South Asian diets in particular are implicated in causing the condition as a result of their high sugar and fat content. Diet is also an important factor in managing this condition, underpinning the need for further evidence about choice of foods and their preparation as well as their social and cultural meanings. A number of research teams [36,53,54] have explored the beliefs of South Asian people who have been diagnosed with type 2 diabetes. However, as Lawton et al. [36] suggest, given the importance of the sharing of food, hospitality and food consumption as part of worship as well as the distributed responsibility for food preparation across several female family members, there is a need for research to investigate social practices and beliefs regarding food and health more broadly. Furthermore, recent research [55] has added to our knowledge of the importance of food practices in the construction of ethnic identities in first and second generation South Asian immigrants living in Canada.

### Research design

In order to explore the relationship between food practices and health in South Asian communities, it was important for us to consider the research design and methods in order, first, to recruit an adequate number of participants from a range of backgrounds; and, second, to avoid collecting one-dimensional data, composed of mainly routine responses that would be perceived as socially acceptable to a researcher, especially a cultural outsider. Collecting high quality and relevant data was crucial to ensure our analysis was able to produce rich descriptions and work towards a more theoretical understanding of how material, social and cultural factors are connected and influence each other in ways that produce effects on health. We paid particular attention to the environment in which data collection was to take place as we were concerned about its effect on participants' responses, and on the relationship and interaction between participants and researchers. We worked on the assumption that where and with whom food is discussed are important considerations in terms of participant engagement and data quality. This view was informed by our previous observations of multidisciplinary clinics for patients with type 2 diabetes, where women's discomfort when being asked to discuss their diet and food with a dietician in a hospital clinic room was clearly visible. We were mindful of the importance of appropriate spaces that could underpin the production of an atmosphere that was relaxed and conducive to talk about food and health. With regard to the relationship and interaction between participants and researchers, we planned to create a safe, facilitative environment which we could achieve by a women-only group of both participants and researchers. To this end, we accessed a wide range of pre-existing groups serving South Asian women in community centres and voluntary or faith-based organisations, such as gardening, cooking and traditional South Asian arts clubs, a language and employment skills centre for women, and a day care facility for South Asian elders. The research team consisted of women from a range of ethnic backgrounds.

In collaboration with a local artist from a South Asian background, we selected a collaborative activity with the aim to mirror food preparation and cooking, thus providing a focus for talk among women. The South Asian craft of Rangoli^1 ^is - like food preparation and cooking - a mainly female activity and shares many of the social and collaborative features of cooking. To enhance this correspondence, the materials used for the Rangoli were dried pulses and grains traditionally used in South Asian cooking. The activity provided a focus for informal conversation and social interaction about food and its role in health, leading to a collaboratively produced piece of artwork that the women could keep. An obvious alternative approach would have been to carry out the data collection in a real cooking session and we would like to consider that in the future. However, there are a number of practical disadvantages including concerns about health and safety and food hygiene which make it a complex activity to plan as a data collection event.

### Recruitment

We made contact with several community groups and registered voluntary organisations, serving South Asian women, through their gatekeepers. We sent written information about the project via email or through the post, and then set up a planning meeting which included whoever our contact at the organisation decided needed to be there. In practice, this was often a volunteer or trustee. The recruitment of the women for the Rangoli data collection sessions was carried out by the project lead in the organisation that had agreed to take part. Our aim was to conduct up to six data collection sessions with 15 to 20 participants in each. We ensured that the project leads were clearly briefed about the project and had written information available. With our help, they produced flyers and posters in the relevant language and style. However, word-of-mouth was the predominant method of recruitment.

### Ethics and consent

The project proposal was reviewed by the university ethics committee. Given that recruitment and data collection took place in community settings, ethical review through the UK National Health Service was not required. Verbal consent was sought at three levels; at the collective level through the lead person of the community group; second, at individual level when the women who had chosen to attend the Rangoli data collection session were welcomed by the lead researcher who reminded them of the purpose of the session, and finally immediately prior to data collection when the digital recorders were switched on.

### Data collection

**Figure 1:**
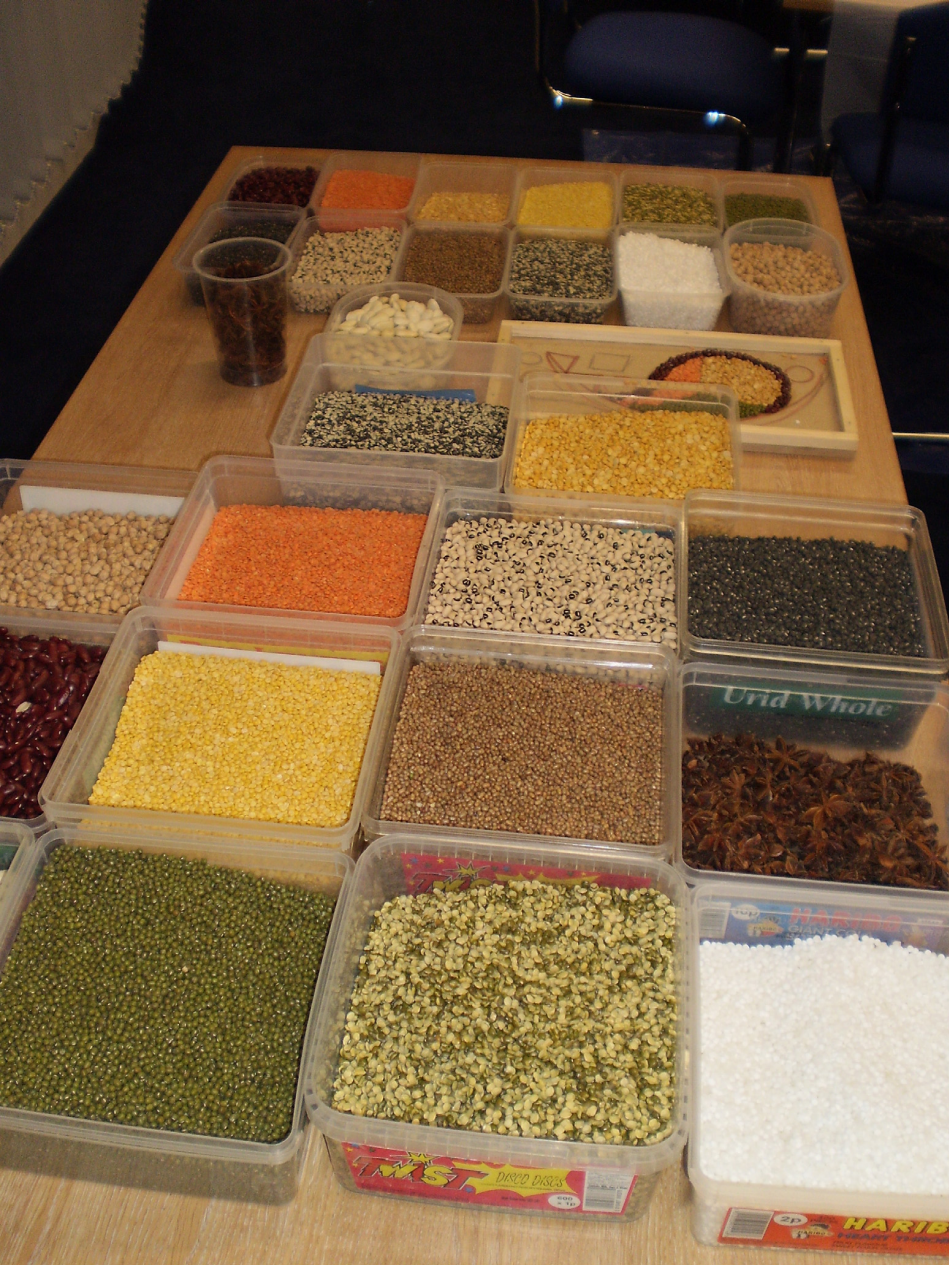
**The use of dried foodstuffs such as dried pulses enhanced the correspondence between food preparation and the South Asian craft of Rangoli**.

**Figure 2:**
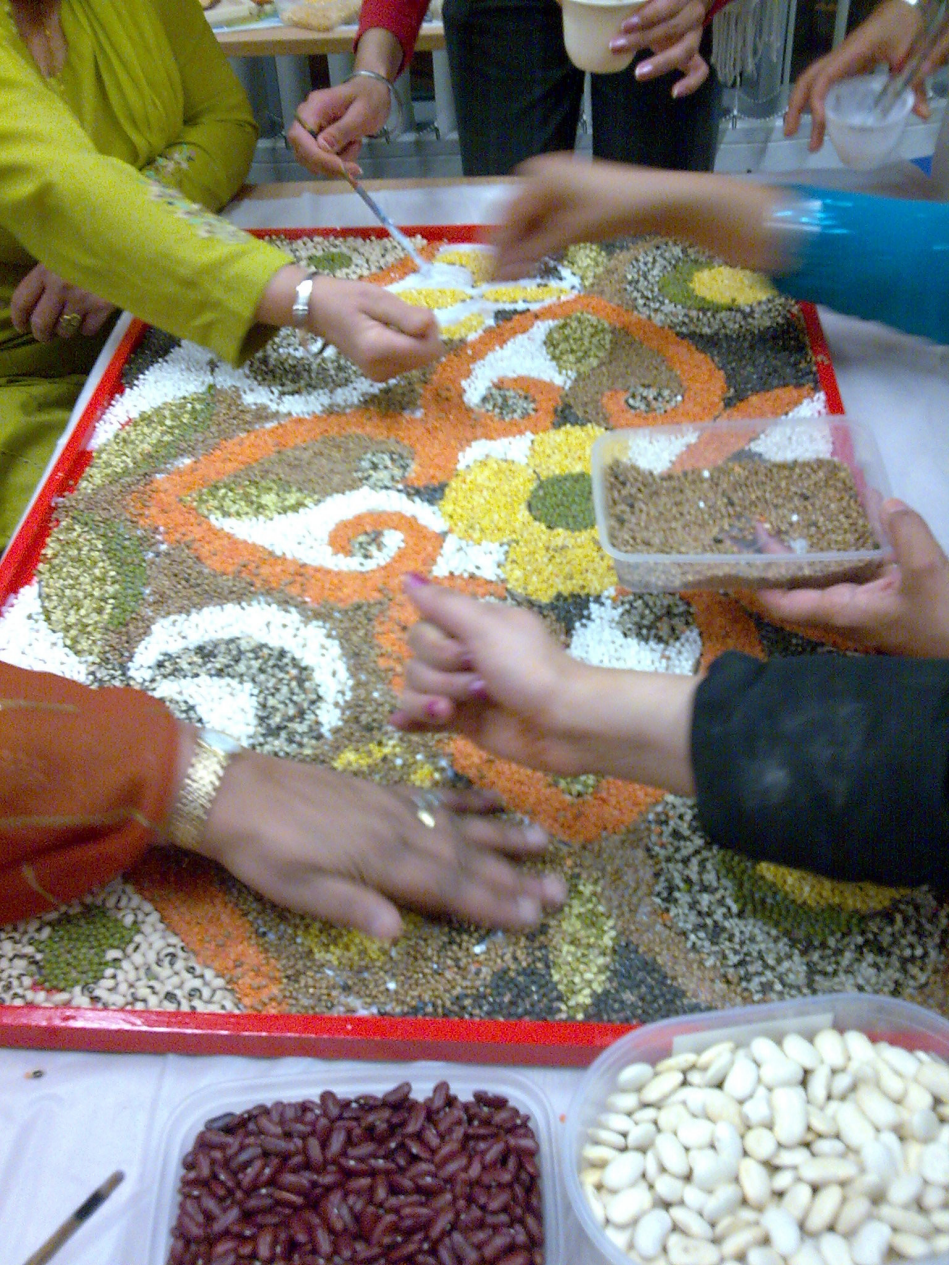
**The research participants worked collaboratively to fill in the Rangoli board while talking to the researchers and each other**.

**Figure 3:**
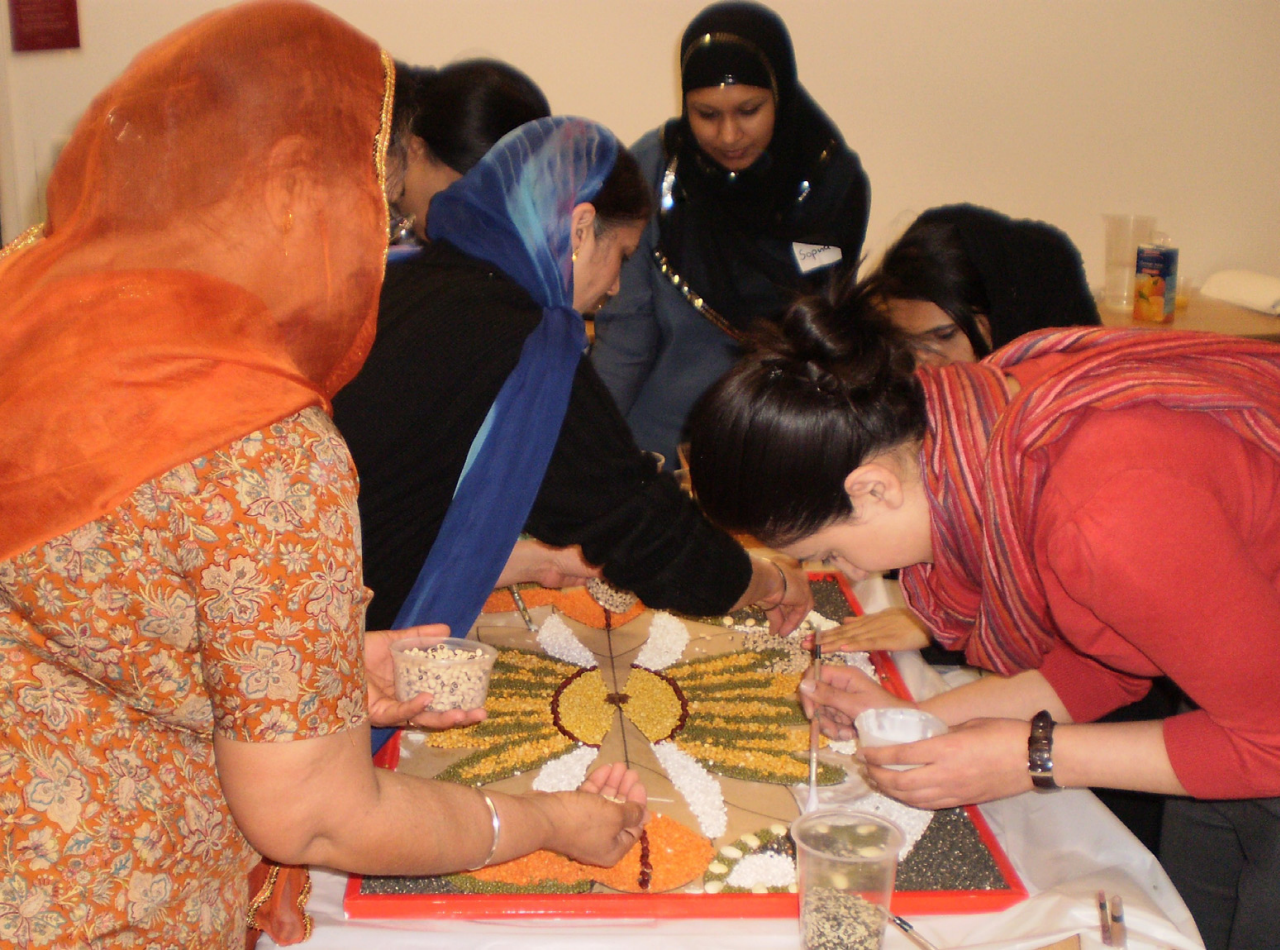
**One of the researchers was taking part in the Rangoli activity while talking to the research participants**.

Each session was facilitated by the artist who led the Rangoli activity while up to six researchers were responsible for the data collection. The room was prepared with two large tables. Each table was set up with one Rangoli board and materials including glue, cereals, pulses, spices, herbs, rice and other dry foodstuffs (Figure 1). Each table accommodated up to twelve women who were seated around it while the artist and researchers moved between the women working at the tables. The artist and researchers introduced themselves as the participants entered the room. Once all expected participants had arrived, the lead researcher opened the session formally with an explanation of the project and our wish to capture in digital audio-recordings the women's views and opinions as they talked to each other and to the researchers. To that end, a digital recorder was placed on each table, and each researcher carried a recorder with her as she moved between the women working at the tables. The role of the artist was to introduce the activity and facilitate the process of constructing the Rangoli (Figure 2 and 3). She demonstrated how to apply the glue on to the board and add the colourful materials to produce the shapes and figures of the Rangoli. Some groups were more skilled than others, as were individuals, and she assisted as necessary to ensure each group of women were able to complete their Rangoli board in around 90 minutes. She responded to requests for information or help, answered questions and also participated in the conversations. The process of the Rangoli construction was flexible and adapted to the level of skill and concentration of the group rather than consisting of a set of reproducible steps. So, for example, the group of women at the South Asian elder care day facility required more assistance from the artist than the group of younger women taking art classes. However, the process of constructing the Rangoli itself was not of prime importance. It merely provided the focus for discussion, the sharing views about food and cooking, the reminiscing and the interactions which were to constitute our data.

All researchers were encouraged to make observational notes, documenting ideas, impressions and insights during the data collection sessions, and any additional information communicated before or after the sessions. These data were shared at the debriefing meetings of approximately two hours, which followed each data collection session. The debriefing meetings were also audio-recorded.

### Data analysis

Analysis was based on the constant comparative method [56,57] in which data collection and data analysis are carried out in parallel. Data were organised using data management software and analysed inductively. The data divided into two broad types - interactive data from discussions among the group, and data obtained through individual interviews in the form of 'off-line' conversations between individual participants and researchers which took place away from the main group. The individual conversations were sometimes initiated by a participant wanting to communicate more information on a particular topic, or on the invitation of a researcher seeking further information on what a participant had contributed in a group discussion. These two types of data, group discussions and individual interviews, were analysed separately, but the findings were later integrated.

## Discussion

As food and diet are implicated in the disproportionate burden that South Asian communities living in the UK bear with regard to chronic illness and type 2 diabetes in particular, we wanted to explore the intersections of faith, culture, health and food in these communities in order to generate evidence for addressing cultural difference in the design and provision of health services. Given that we were interested in the voices of South Asian women many of whom are largely invisible in public life on account of their ethnicity, gender and family structures and who are rarely represented in research, it was important to select an approach to data collection which was culturally in tune with women's lives and sensitive to their values and beliefs. The aim was to facilitate the kind of talk through which lay knowledge and beliefs are constructed and communicated, avoiding responses which participants believe are the ones researchers want to hear and which reflect public health messages rather than the 'lay theories' [58] that are embedded in daily routines and cooking practices. The attention to cultural sensitivity, the notion that meaningful research must be founded on trust and commitment and on the ongoing relationship between researchers and communities are features which are shared with a number of approaches to research with seldom heard communities [18]. However, the programme of research under the auspices of which this project was undertaken did not envision the type of community involvement in data collection, analysis and the generation of findings characteristic of Participatory Action Research [59] or Community Based Participatory Research [60]. Instead the focus of this study was the production of data rich in detail of social and cultural factors that may not have been generated through semi-structured interviews.

### Reflections on participant recruitment and ethics

The Rangoli activity which provided the focus for the data collection was led by a visual artist who had previous experience of working within the health field. She has a South Asian background and uses her cultural and religious roots in her art. The lead researcher is a female health professional of white European descent and works in the discipline of medical sociology. Both were assisted by three female bicultural researchers of South Asian origin, one with a background in biomedical science and two in sociology. All were involved in the recruitment of participants which was carried out through a combination of personal contacts and word-of-mouth, desktop research to identify third sector organisations, and what we called 'shoe leather' research. This involved travelling in the local area and 'walking the patch', and consisted of meeting potential gatekeepers to appropriate women's groups at local events and open days, attending public events, speaking to people about our work informally, meeting community workers in the field, and generally raising our profile as researchers in the community. Such efforts were time consuming and outcomes were not easily defined in advance. They also did not always yield direct results in terms of recruitment. We found it particularly difficult to access Pakistani Muslim women. A cultural insider explained that this might be due to families' unwillingness to discuss what are deemed to be private family matters, namely the preparation and eating of food and the social and religious practices surrounding it, with strangers and for a public purpose, i.e. research. However, our outreach activities were very helpful in building relationships and in demonstrating our personal as well as professional commitment to ensuring that the research led to benefits for women in those communities. This phase of the project lasted around three months, but coincided with the first data collection sessions. We were able to invite a project lead, who was considering hosting a data collection session, to one that had already been organised to enable her to decide whether her group would be prepared to participate. In terms of success in recruitment, personal, family and community contacts by the members of the research team with a South Asian background were the most fruitful, followed by contacts with local faith organisations. We found that many community organisations that served the needs of women were organised around faith groups which in the Birmingham area are Sikh, Muslim and Hindu, and that group leads were receptive to activities promoting women's health and wellbeing. We were able to build trust with the group leads through our shared concerns about health and well-being in their communities.

Building relationships with gatekeepers and organisational or groups leads was essential in legitimising our work to women who were interested in participating in our project. When we had a positive response from a group lead, we arranged a meeting at the group's premises or community venue which was attended by the artist, the lead researcher and by one or two of the researchers. These meetings were crucial in terms of, first, building trust and confidence in the research team and the artist and, second, organising the data collection event and addressing the practicalities for potential participants. We stressed repeatedly that the event was to be cost-neutral to the group or organisation as there was anxiety over current and future funding. We also underlined that costs for room hire, materials, refreshments, travel and child care expenses would be met by the research team. The timing of the event needed to be carefully worked out with each group in order to reduce the burden of participation in the project and to ensure that as many women as possible were able to attend.

We avoided formal written consent procedures because of their potential to exclude people from participation in research. Our rationale for the decision not to use formal procedures was that we were planning to recruit a seldom heard group that is marginalised at many levels: they are part of a migrant community that tends towards collectivist rather than individualistic values [61]; as women in their cultural group they are subject to patriarchal social control; the older generation of women tend to have fewer written language skills and also do not necessarily subscribe to the primacy of written consent [62]. Furthermore, formal consent procedures requiring a signature would have obliged some women to ask their husband or male relative to sign on their behalf, a requirement which could have discouraged women from taking part. Participation may also have been refused by a husband or male relative. By keeping the project informal and part of the usual activities within their community group, we anticipated that women would be encouraged to take part. In running six data collection session over 12 months, we recruited over 120 women. Not everyone who participated in the Rangoli activity was able to contribute significantly to the data, but the majority added at least some individual or group data.

We had been concerned that some women might be uncomfortable about audio-recordings and either decline to participate or refuse to be audio-recorded which would have required the removal of the recorder from their table. We were prepared for one member of the research team to take notes instead. However, none of the women refused audio-recording either in the whole group, or when we asked them individually. 'You give us Rangoli, we give you talk' was how one of the participants expressed her satisfaction with the reciprocal arrangement we had made. We also wanted to take some photographs of the session as it progressed, focusing on the women's hands as they handled the materials and built up the Rangoli patters. Some of the women requested not to have their faces photographed, but were happy for their hands to be included. Consent has been given for the publication of the photographs accompanying this article. One of the researchers spoke to each of the women to collect anonymous demographic data including age range; country of origin or parents' country of origin; whether they had been given a diagnosis of type 2 diabetes or pre-diabetes; and whether they had family members diagnosed with the condition, and if so, how they were related and if they lived in the same household. We used several digital recorders: one was placed on each of the tables around which the Rangoli activity took place, and each researcher carried one.

Contrary to our previous experience of running focus groups, we found that many more women turned up to the data collection sessions than had originally signed up. This was the result of last minute invitations to female family members or neighbours who had heard about the session and wanted to come along. Thus the data collection sessions were social occasions for the women, reinforced by the hospitality arranged by the research team so that data collection sessions taking place in the evening were preceded by a light supper whereas those taking place in the morning finished with lunch. Water, juice and hot drinks were available throughout the sessions. The hospitality contributed to a convivial, relaxed atmosphere.

### Reflections on data and language

As a result of the interactive nature of the data collection technique, the data we collected were heterogeneous and very different to those gathered through semi-structured interviews. The artist created a relaxed, sometimes noisy and exuberant atmosphere which encouraged women to talk to each other and to the researchers. This presented a challenge to audio-recording as the background noise led to problems with the sound quality which made transcribing the data difficult. Different languages and dialects being spoken also added another layer of complexity to the data. Most of the women spoke to the researchers in English although they would speak to each other in their mother tongue. The first generation women struggled sometimes with their English language skills, but because they found themselves in an all-female, informal environment, they wanted to speak to the white researchers in English. The younger women spoke English to the researchers and well as to each other.

Some direct translation was possible after the sessions on listening to recordings and transcribing the data, when researchers with the necessary language skills were able to translate verbal interactions between the women, or between a participant and researcher. Sometimes daughters interpreted for their mothers during the sessions, and in one group the centre lead and second generation women working for the organisation interpreted researcher questions into the participant's native language and the participant responses back into English. However, we did not check for the accuracy or the conceptual equivalence [63] of the translations provided by these lay translators. We also did not account for the effects of translators on the data. This is a methodological limitation that needs to be addressed in this kind of study in order to increase the what has been termed '"cross-language trustworthiness" which shows that the researcher systematically accounted for factors that would compromise the credibility [and] transferability ... of translated data [[64]; p. 285].

Some data were unusable due to the poor sound quality as a result of background noise whereas other data were clearly not related to the research question. The latter included conversations about the technical aspects of the Rangoli activity, such as how much glue to use or what patterns to fill in next, and included private talk about social events or family business which was captured by the microphone, but was clearly not intended for research use.

Apart from the observational data which we drew on to shed light on the social, cultural and community context, all other were digitally recorded. The diversity of data with regard to speakers and interactions taking place, made data transcription, management and analysis challenging and required some decisions about the data in order to organise and reduce them and also make them amenable to systematic analysis.

### Reflections on data quality and analysis

We began our thematic analysis as we transcribed and organised the transcripts from the data collection sessions, also including notes and transcripts from the researcher debriefing sessions. This resulted in a complex and heterogonous data set which presented us with analytic challenges and surprises. We had divided the data into two broad types - group/individual interactions and one-to-one interviews. Interactive data were obtained through (1) direct responses to direct questions and prompts from researchers to the group involved in Rangoli activity; (2) interactive talk between women; (3) interactive talk between women and researchers. These data were similar to that of focus group data, but different in two fundamental ways: in the Rangoli setting, women rarely spoke more than a sentence or two in one turn, and interruptions occurred frequently because the activity demanded participants' attention and collaboration. The result of these short interactive data sequences was what has been termed 'small stories' [65-67]. 'Small stories' focus on the stories we tell each other in passing, in our everyday encounters with each other; they are 'narratives-in-interaction' [[68]; p. 235]. They refer to material that is not neatly storied, but fragmented and sometimes even incoherent. They arise in everyday social interactions and reflect particular local practices [65]. The excerpt below gives an illustration of two interwoven and familiar 'small stories' about type 2 diabetes: one story concerns a destiny out of the individual's control; the other is one of prevention, diet and exercise.

Woman 1: You can't ever cure it, can you? It's true though, isn't it?

Woman 2: What's not curable?

Woman 1: Sugar. Diabetes.

Woman 2: You can prevent it.

Woman 1: You can't prevent it if it runs through your family.

Woman 3: No, you can prevent it.

Woman 1: Not if your parents and your uncles got it and if your aunts have got it, everyone in your family has got it, then how can you prevent it?

Woman 3: You can prevent it. You can diet and exercise.

Woman 1: I know the healthiest people and they've got diabetes. It's their fate, isn't it?

'Big stories', on the other hand, emerged from the more narrative data derived from the 'off-line' conversations between individual participants and researchers which took place away from the main group. The data they yielded resembled conventional individual interview data, but as they were collected within the social context of the Rangoli session, frequent reference was made to what had been said previously in the group situation, or expanded upon what had already been mentioned. This data set included stories about the experiences of parents' migration, the effects of those experiences on their community and on second generation South Asians in the UK. The stories were often constructed with what Morse [[69]; p. 3] has called 'shadowed data'. These are participants' discussions of not only their own experiences, but also of those of others. In this instance, participants relayed their interpretation of the experiences of their parents and those of their parents' generation, how their own experiences differ from theirs, and for what reasons. The young woman speaking here gives an account of her parents' migration and identifies their changing attitude to food and traditional eating practices:

At the beginning they worked really hard and they just settled down to fit in. So they worked their socks off to do that an, you know, when they started to make it then they realised that actually, we have a house, we have a job, and then they just chilled out. Then it was just like the family would come round and just eat and drink because they realised that they made it, that they were here ... they didn't have to worry about anything now, but they didn't realise the food they were eating then like ten, twenty years down the line was affecting their health. (...) It was a big thing for them to come here and it took 100 per cent out of them to come here and to take like all of the racism and to take all the headaches, you know, and the rubbish jobs they had to do when they came here. But then they made it and I think it was just a matter of finding "oh we can have this food and there are these restaurants and fast food now so you don't have to cook at home; why would you cook at home, why would you want to eat this basic Daal when you can have things like pizza and take-away?"

These stories, both 'big' and 'small', have been helpful in elucidating the themes that have been generated through the constant comparative analysis of data collected, both within and across data collection sites and groups. One of these themes which is most relevant to producing evidence for the design of future health services is that of the relationship between food, social practices and religion, and women's pivotal position in relation to all three. What the 'small stories' in particular were able to contribute to the development of this theme were the connections between food, and social and moral responsibilities: much of women's roles in the preparation of food was not just about technical skill and social duty, but also about their moral worth as women, wives and mothers in nourishing their family with health giving food. Honouring traditions handed down through generations of women was seen as a social as well as a moral obligation. In some instances, these obligations were also linked to religious practice. The women's accounts suggested that they are enmeshed in family and social structures which define who they are and how they prepare food. This indicates that what, why and how food is prepared is a deeply complex social and cultural phenomenon that is unlikely to be amenable to educational interventions which seek to address a biomedical knowledge deficit and aim to promote self-efficacy.

We propose that the use of techniques that are specifically tailored to attend not only to the research questions or objectives, but also to the values and cultural needs of the participants, has the potential to generate finely grained and nuanced data which is not just theoretically interesting or helpful in building descriptive categories. Rather, such culturally responsive methods also have the potential to increase the explanatory power of study results. Indeed, the findings from this project so far suggest reasons why educational interventions in isolation may be ineffective, and why strategies to engage South Asian women in educational sessions have met with limited success [35]. These reasons are likely to be related to a mismatch between the conceptual underpinnings of educational intervention and the way women construct their identities as social and moral members of a family and community. Such findings have the potential to generate highly practical and applicable evidence for informing the design of culturally sensitive health care services.

## Summary

The exclusion from health research of groups most affected by poor health is an issue not only of poor science, but also of ethics and social justice. Even if exclusion is inadvertent and unplanned, policy makers will be uninformed by the data and experiences of these groups. The effect on the allocation of resources is likely to be an exacerbation of health inequalities. We have argued that the methods traditionally used in health research requiring formal recruitment strategies and written consent procedures, as well as semi-structured interviews which are the most commonly used method in qualitative health research, not only serve to deter some groups and communities from taking part in health research, rendering them what has been termed 'hard to reach', but limit the potential for producing high quality and rich data. By sharing our experience of carrying out a research project to explore the intersections of faith, culture, health and food in these communities to generate evidence for addressing cultural difference in the design and provision of health services, we have emphasised the importance of building trust and working in culturally sensitive ways in order to be inclusive in our research practices. These are particularly relevant in relation to recruitment and data collection. Adopting culturally sensitive methods can result in complex and heterogeneous data sets which present challenges for analysis and interpretation. Yet they also have the potential to produce finely grained and contextually pertinent findings, bringing to the surface social and cultural factors which are not obvious to cultural outsiders. Ensuring opportunities for research team members from varied cultural backgrounds to reflect upon those aspects of the data that express or contain (explicit or implicit) cultural assumptions further enhances the analysis process. The analytic challenge for qualitative health researchers engaging with such methods is to work with the narrative repertoires such data uncover, and propose ways in which they can be mobilised in the development of culturally responsive services and interventions, in the communication practices of health care professionals, and in the design of health information and education.

## Competing interests

The authors declare that they have no competing interests.

## Authors' contributions

SR and SG designed the study. SR carried out data collection and analysis. NG contributed to data collection, analysis, and interpretation. SR drafted the original manuscript. All authors have been involved in revising it critically for intellectual content, and have given final approval of the version to be published.

## Notes

Rangoli is a popular and decorative South Asian art form, similar to mosaics, through which pictures are made out of rows of colourful materials, often in intricate geometrical patterns. They are temporary pieces of art usually created on the ground with flower petals or coloured chalk to bless a house and its inhabitants, and they are later swept up. Their significance is one of bringing peace and harmony. In order to reinforce the connection with food we supplied dried cereals, pulses, spices, herbs, rice and other dry foodstuffs as materials for the Rangoli activity. We used large boards as bases onto which the dry foodstuffs could be glued which meant that the Rangoli mosaics were permanent and could be displayed in the community setting after the researchers had left.

## Pre-publication history

The pre-publication history for this paper can be accessed here:

http://www.biomedcentral.com/1471-2288/12/7/prepub
